# Proteomic study reveals a functional network of cancer markers in the G1-Stage of the breast cancer cell cycle

**DOI:** 10.1186/1471-2407-14-710

**Published:** 2014-09-24

**Authors:** Milagros J Tenga, Iulia M Lazar

**Affiliations:** Department of Biological Sciences, Virginia Polytechnic Institute and State University, 1981 Kraft Drive, Blacksburg, VA 24061 USA

**Keywords:** Cell cycle, Cancer markers, Proteomics, Mass spectrometry

## Abstract

**Background:**

Cancer cells are characterized by a deregulated cell cycle that facilitates abnormal proliferation by allowing cells to by-pass tightly regulated molecular checkpoints such as the G1/S restriction point. To facilitate early diagnosis and the identification of new drug targets, current research efforts focus on studies that could lead to the development of protein panels that collectively can improve the effectiveness of our response to the detection of a life-threatening disease.

**Methods:**

Estrogen-responsive MCF-7 cells were cultured and arrested by serum deprivation in the G1-stage of the cell cycle, and fractionated into nuclear and cytoplasmic fractions. The protein extracts were trypsinized and analyzed by liquid chromatography - mass spectrometry (MS), and the data were interpreted with the Thermo Electron Bioworks software. Biological characterization of the data, selection of cancer markers, and identification of protein interaction networks was accomplished with a combination of bioinformatics tools provided by GoMiner, DAVID and STRING.

**Results:**

The objective of this work was to explore via MS proteomic profiling technologies and bioinformatics data mining whether randomly identified cancer markers can be associated with the G1-stage of the cell cycle, i.e., the stage in which cancer cells differ most from normal cells, and whether any functional networks can be identified between these markers and placed in the broader context of cell regulatory pathways. The study enabled the identification of over 2000 proteins and 153 cancer markers, and revealed for the first time that the G1-stage of the cell cycle is not only a rich source of cancer markers, but also a host to an intricate network of functional relationships within the majority of these markers. Three major clusters of interacting proteins emerged: (a) signaling, (b) DNA repair, and (c) oxidative phosphorylation.

**Conclusions:**

The identification of cancer marker regulatory components that act not alone, but within networks, represents an invaluable resource for elucidating the moxlecular mechanisms that govern the uncontrolled proliferation of cancer cells, as well as for catalyzing the development of protein panels with biomarker and drug target potential, screening tests with improved sensitivity and specificity, and novel cancer therapies aimed at pursuing multiple drug targets.

**Electronic supplementary material:**

The online version of this article (doi:10.1186/1471-2407-14-710) contains supplementary material, which is available to authorized users.

## Background

Howard and Pelc described four consecutive phases of the cell cycle: G1, S, G2 and M [1]. Each phase needs to be completed before the next one can proceed: the G1-phase is a period of growth in preparation for replication, the S-phase a period in which the DNA content is duplicated, the G2-phase a period of growth in preparation for mitosis, and the M-phase a period in which the cell divides into two identical daughter cells. Multiple regulatory events, termed checkpoints, verify whether certain cellular processes have occurred properly before allowing the cells to proceed from one phase to another [2–6]. For example, DNA damage checkpoints at the G1/S and G2/M transition boundaries, and spindle checkpoints during the M-phase, have been recognized. These checkpoints allow either for DNA repair, or correct chromosome alignment on the mitotic spindle, respectively, before the next steps of the cell cycle can proceed. In 1978, Pardee described the G1/S restriction point (R-point) as an essential regulatory event in the G1-phase [3]. The period before the R-point is uniquely sensitive to growth factor stimulation, and in the absence of mitogenic signaling, normal cells exit the cell cycle and enter a reversible dormant/quiescent state termed G0. Alternatively, in the presence of major perturbations in the cell cycle regulatory machinery, such as DNA damage, normal cells attempt to repair such damage, or in the case of failure, commit apoptosis. Unlike normal cells, cancer cells evolved the ability to evade the restriction point and continue through the cell cycle even if DNA damage is detected. After the R-point, both normal and cancer cells are unaffected by the removal of growth factors or other deregulatory events, and enter the S-phase, committing to a round of cell division. Therefore, the R-point emerges as the most critical point in cell cycle control. Key to its regulation is the phosphorylation of the retinoblastoma protein (pRb or RB1) by active cyclin D-CDK4/6 and cyclin E-CDK2 complexes in early and late G1, respectively, an event that results in the release of E2F transcription factors that signal the cell to continue into the S-phase, replicate and proliferate. Hundreds of E2F target genes that are involved in DNA replication and cell cycle signaling, as well as DNA damage repair, programmed cell death, development and cell differentiation, have been identified. Traditional molecular biology and biochemistry approaches have greatly contributed to understanding breast cancer cell cycle regulation. However, the introduction of high-throughput genomic and proteomic methods, and the escalating development of novel bioinformatics tools, have revolutionized cancer research. Large lists of genes and proteins involved in important biological processes are generated with the aim of providing a comprehensive picture of all concurring events in a cell, the challenge continuing to rest with the interpretation of such voluminous data. As the mechanisms used by cancer cells to escape the R-restriction point continue to remain unclear, the objective of this study was to use mass spectrometry technologies to generate a comprehensive map of proteins that are expressed in the critical G1-stage of the cell cycle in a representative model system of ER + breast cancer such as MCF-7, and make use of bioinformatics tools to explore: (a) whether cancer marker proteins reported by previous studies (rather unrelated) can be associated with this stage of the cell cycle, (b) whether a particular subcellular localization is characteristic for these proteins, and (c) whether these markers form regulatory networks that promote cell proliferation and can be placed in a broader context of cancer-relevant functional roles to advance a panel with biomarker and drug target potential.

## Methods

### Cell processing

MCF-7 cells (ATCC, Manassas, VA) were grown in EMEM with 10% FBS and 10 μg/mL bovine insulin, in an incubator at 37°C with 5% CO_2_ [7, 8]. The cells were arrested in the G1-phase by serum-deprivation for 48 h, in a medium consisting of DMEM and 4 mM L-glutamine, harvested, separated into nuclear and cytoplasmic fractions (Cell Lytic™ NuCLEAR™ extraction kit, Sigma, St. Louis, MO), digested with trypsin (Promega Corporation (Madison, WI) at 37°C for 24 h (50:1 substrate:enzyme ratio), and analyzed by nano-liquid chromatography (LC)-MS/MS with a linear trap quadrupole (LTQ/Thermo Electron Corporation, San Jose, CA) mass spectrometer. FACS analysis was performed with a Beckman Coulter EPICS XL-MCL analyzer (Brea, CA, USA). The protein content was measured by the Bradford assay on a SmartSpec Plus spectrophotometer (Bio-Rad, Hercules, CA). The sample analyzed by MS contained 2 μg/μL MCF-7 proteins. LC separations were performed with an Agilent 1100 LC system (Palo Alto, CA) and in-house prepared nano-separation columns (100 μm i.d. x 12 cm) packed with 5 μm Zorbax SB-C18 particles. Common reagents were purchased from Sigma, cell culture media from ATCC and Invitrogen (Carlsbad, CA), and HPLC-solvents from Fisher Scientific (Fair Lawn, NJ). Sample preparation and LC-MS/MS analysis protocols were described in detail in previous manuscripts [7, 8].

### Data processing

A minimally redundant *Homo sapiens* protein database from SwissProt (2008/40,009 entries) and the Bioworks 3.3 software (Thermo Electron) were used for protein identifications. Conditions for peptide selection included: only fully tryptic fragments with maximum two missed cleavages, no posttranslational modifications, peptide and fragment ion tolerances set at 2 amu and 1 amu, respectively,% fragment ion coverage >30% (from any combination of theoretical b, y and a ions), all peptides matched to unique proteins in the database, and Sequest Xcorr vs. charge state parameters set at 1.9, 2.2 and 3.8 for singly, doubly and triply charged peptides, respectively. At the protein level, the Bioworks p-score threshold was set at ≤0.001. Proteins matched by one unique peptide were considered only when could be identified in at least two biological states or replicates. A few proteins matched by a single peptide count were allowed in the analysis, due to their relevance, but the associated SwissProt IDs should be treated in such cases with prudence due to the possibility of existing protein isoforms that share the same peptide. The peptide p-values were for these cases < 0.001. False discovery rates (FDR) were determined by searching the raw data against a forward-reversed protein sequence database. FDRs were <3% and <1% at the protein and peptide levels, respectively. Specific parameter settings for the use of bioinformatics tools were: GoMiner included all evidence codes; STRING parameters were set to high confidence, ≤10 interactors, network depth 1 and all active prediction methods; the DAVID enrichment p-score threshold was 1.3 (shown as -log transformed value), with a *Homo sapiens* background and classification stringency set to medium.

## Results and discussion

### Sample and data analysis

**Figure 1:**
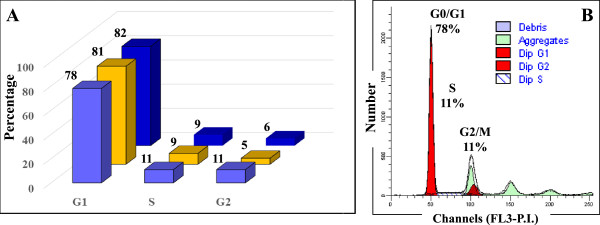
**FACS analysis of MCF-7 cells. (A)** Bar graph illustrating the reproducibility of cell cycle arrest in G1 by serum deprivation for 48 hours in three biological replicate cultures; **(B)** FACS diagram of G1-stage MCF-7 cells.

**Figure 2:**
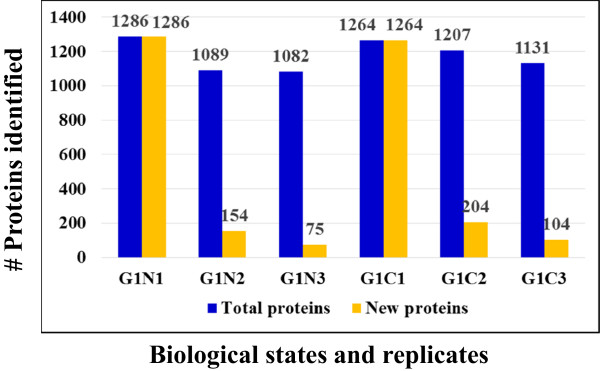
**Reproducibility of protein identifications in three biological replicates of G1N/G1C MCF-7 cells.**

**Figure 3:**
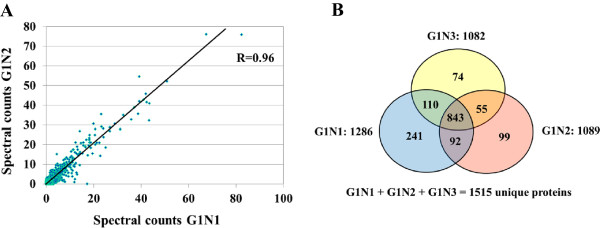
**Protein overlaps among biological replicates of MCF-7 cells. (A)** Scatter plot of protein identifications in two biological replicates of MCF-7 G1N cells (G1N1 vs. G1N2, total 1239 proteins). **(B)** Venn diagram of protein overlaps between three biological replicates of MCF-7 G1N cells.

While the key events of the cell cycle control take place in the nucleus, a number of relevant signaling pathways activated by mitogenic stimuli proceed through the cytoplasm, prior to impacting the nuclear sequence of events. Furthermore, many proteins are shuttled between the cytoplasm and nucleus as a means of functional activation/deactivation. To increase the number of identifiable proteins and generate a comprehensive map of the biological processes that unfold in the G1-stage of the cell cycle, the MCF-7 cells were separated into nuclear and cytoplasmic fractions. Three biological replicates were prepared to enable a confident selection of identifiable proteins, and five LC-MS/MS technical replicates were performed to maximize the number of identifiable proteins and the number of spectral counts per protein [7, 8]. A total of six samples were generated from the two cell states [i.e., G1-phase nuclear (G1N1, G1N2 and G1N3) and G1-phase cytoplasmic (G1C1, G1C2 and G1C3)] and a total of 30 LC-MS/MS analyses were performed. Reproducibility was assessed at every step of the analysis. The cell cycle distribution in each cell culture was evaluated by flow cytometry (Figure 1). The arrested cells were found primarily in G1 (~81%), and only a small proportion in S (~10%) and G2 (~7%), respectively. A bar graph illustrating the trend in protein identifications is provided in Figure 2. Each biological replicate displays cumulative protein identifications in 5 LC-MS/MS analyses, and new protein identifications relative to the previous replicates. After MS data processing and filtering, in-house developed Perl-scripts were used for aligning protein and peptide spectral count data [9]. A total of 2375 proteins were identified, of which, 2000 with two or more spectral counts. The average number of identified proteins and matching counts in each of the 6 cell states was 1176 (CV = 7.5%) and 4030 (CV = 8.9%), respectively, with a total of 1515 proteins in the combined nuclear fractions and 1572 in the combined cytoplasmic fractions. The correlation coefficient of protein identifications based on spectral count data in any two biological replicates of a cell state reached values as high as R = 0.96, as shown in a representative comparison involving the G1N1 and G1N2 fractions containing a total of 1239 proteins (Figure 3A). As expected, however, due to biological and technical variability, the effective overlap of protein IDs between all three replicates did not exceed ~75% (Figure 3B). Nevertheless, the above described workflow enabled the identification of a sufficient number of proteins for extracting meaningful biological information despite the lack of a high-end mass spectrometer platform for performing the experiments. The ExPASy Proteomics Server [10], GoMiner [11], DAVID Bioinformatics Resources [12, 13], STRING functional protein association networks 8.3 [14] and the GeneCards [15] bioinformatics tools were used for the functional interpretation of the data. GoMiner analysis revealed that the nuclear cell fractions comprised 57–62% and 59–64% proteins with nuclear and cytoplasmic categorization, respectively. The cytoplasmic fractions comprised primarily cytoplasmic proteins (83–84%), and only a small fraction of nuclear proteins (32–33%). While complete separation of the two cell fractions was not experimentally achievable, the nuclear enrichment process resulted in an increase of the nuclear proteins from 15–20% in a whole cell extract, to >50% in the nuclear-enriched fraction. DAVID functional clustering of the MCF-7 proteins with two or more spectral counts returned over 150 clusters with enrichment scores > 1.3. The large number of clusters reflects that the dataset is representative of a broad range of basic biological processes that occur in a cell. The top scoring clusters included processes related to the biosynthesis and processing of nucleotides, RNA, proteins and ATP, and to transport, proteasome and metabolism. Additional file 1 lists the identified proteins, their total spectral count, and their identification in the nuclear or cytoplasmic fractions. The overlapping nuclear/cytoplasmic categories (i.e., ~30%) included proteins with roles in gene expression/translation/protein biosynthesis, glycolysis, glucose/carbohydrate metabolism, and intracellular transport.

### Query for putative cancer markers

**Figure 4:**
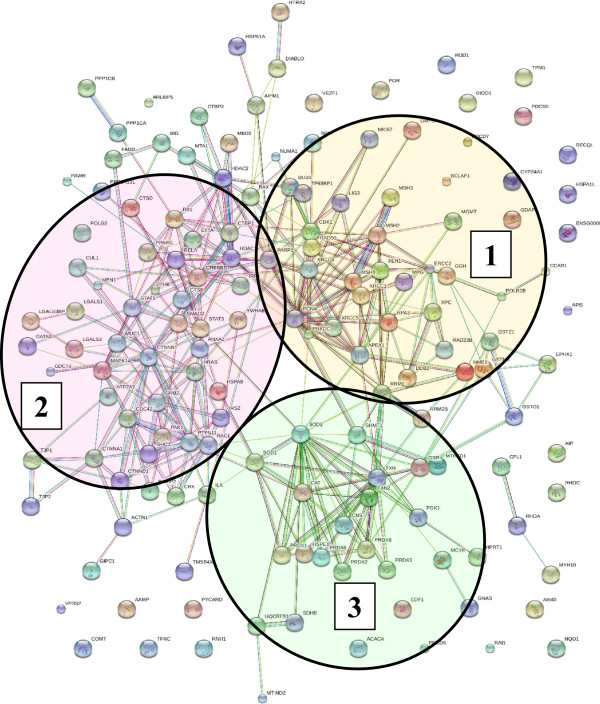
**STRING functional association network of 153 G1-stage cancer markers: (1) maintenance of genome integrity/DNA repair; (2) signaling/cell cycle regulation; (3) energy production/metabolism/stress/oxidative phosphorylation.**

Overall, from the list of 2375 proteins, GoMiner/DAVID categorization returned a considerable number of proteins involved in biological processes representative of all hallmarks of cancer [6, 16] that matched multiple pathways in the Kegg cancer diagram, i.e., proliferation, cell cycle, apoptosis, evasion of apoptosis, failed repair of genes, insensitivity to growth factors, sustained angiogenesis, and PPAR signaling [17]. The list of 2375 was queried for the presence of proteins with role in cancer development or with previously reported biomarker potential. The search in the DAVID disease database returned a list of 96 proteins associated with cancer, of which 51 proteins were matched to breast cancer. Table 1 enlists the identified markers and spectral count data, categorized according to biological processes of relevance to cancer. Additional file 2 enlists supplemental information such as associated GO biological processes, GO cellular compartments, GO molecular functions, Kegg pathways and associated diseases, STRING descriptions, full Sequest report and gene abbreviations. Table 1 was complemented with a set of 57 G1-stage proteins with known significance to cell cycle regulation and cancer [6, 12, 15], shown as entries in *italic*, to amount to a total of 153 protein I.D.s.Proteins of relevance to cancer, but not identified in the dataset, were included in the discussion, but were not included in the generation of lists, figures or the STRING diagrams. Cancer-relevant proteins were present in both nuclear (100 proteins) and cytoplasmic (102 proteins) fractions. Most importantly, a STRING protein-protein interaction diagram revealed a widespread connectivity between these randomly mapped cancer proteins, and redundant identification of the same categories with relevance to cell cycle regulation and proliferation, suggesting the possibility of a useful biomarker panel for diagnostic purposes, or of novel drug candidates that could be targeted synergistically in cancer therapy (Figure 4). Three main networks emerged from the list: (1) signaling and cell cycle regulation, (2) maintenance of genome integrity and DNA repair, and (3) oxidative phosphorylation, stress, energy production and metabolism. While the advocated protein panel is not necessarily specific to MCF-7, and while differential expression profiling was not the purpose of the present study, preliminary comparisons to non-tumorigenic G1-arrested MCF-10 cells confirmed that roughly two thirds of the MCF-7 markers changed spectral counts more than 2-fold, and some even more than 10-fold, when comparted to MCF-10. The results also confirm that proteomic analysis of relevant cancerous cell states can capture in a single experiment protein panels that previously could be identified only by multiple studies, with various model systems, and using various biochemical/biological approaches and tools. A subset of proteins displayed either very small, or, essentially, no change in spectral counts (APEX1, KU70/KU86, LEG3, PARP1, PGK1, PHB, PRDX2, PRKDC, RAC1, RHOA/RHOC, SHC1, TBB3/TBB5, TYB4, UCRI, ZO). Future work will discuss in detail the quantitative comparison of the two cell lines in both nuclear and cytoplasmic fractions. The functional relevance of the most prominent protein clusters that were identified within the three major categories, as well as their broader impact on cancer cell proliferation is discussed below.

**Table 1:** **Biological categorization of MCF-7 proteins matched in the DAVID disease/cancer database (entries in** ***italic*** **are not all cancer markers, but were included in the list due to their functional relevance to the marker proteins)**

ID	Protein name	MW	Total counts	Cancer	Breast cancer
**Cell cycle/division/check-point/proliferation**					
O43684	BUB3_HUMAN Mitotic checkpoint protein BUB3	37131.2	78	Cancer	Breast cancer
P06400	RB_HUMAN Retinoblastoma-associated protein	106091.7	15	Cancer	Breast cancer
P12004	PCNA_HUMAN Proliferating cell nuclear antigen	28750.3	17	Cancer	
P35232	PHB_HUMAN Prohibitin	29785.9	96	Cancer	Breast cancer
P78527	PRKDC_HUMAN DNA-dependent protein kinase catalytic subunit	468786.9	889	Cancer	Breast cancer
P07437	TBB5_HUMAN Tubulin beta chain	49639	230	Cancer	Breast cancer
Q13509	TBB3_HUMAN Tubulin beta-3 chain	50400.3	127	Cancer	Breast cancer
Q14980	NUMA1_HUMAN Nuclear mitotic apparatus protein 1	238113.2	505	Cancer	Breast cancer
Q12888	TP53B_HUMAN Tumor suppressor p53-binding protein 1	213440.8	7	Cancer	Breast cancer
Q6P1J9	CDC73_HUMAN Parafibromin	60539.2	12	Cancer	
P37231	PPARG_HUMAN Peroxisome proliferator-activated receptor gamma	57583.2	5	Cancer	Breast cancer
P46013	*KI67_HUMAN Antigen KI-67*	358471.9	50		
P31947	*1433S_HUMAN 14-3-3 protein sigma*	27756.7	67		
Q8IX12	*CCAR1_HUMAN Cell division cycle and apoptosis regulator protein 1*	132738.6	72		
P06493	*CDC2_HUMAN Cell division control protein 2 homolog*	34073.9	20		
P62136	*PP1A_HUMAN Serine/threonine-protein phosphatase PP1-alpha catalytic subunit*	37487.8	179		
P62140	*PP1B_HUMAN Serine/threonine-protein phosphatase PP1-beta catalytic subunit*	37162.6	9		
Q13616	*CUL1_HUMAN Cullin-1*	89622	12		
Q13547	*HDAC1_HUMAN Histone deacetylase 1*	55067.8	120		
Q92769	*HDAC2_HUMAN Histone deacetylase 2*	55328.8	31		
**Apoptosis**					
O14737	PDCD5_HUMAN Programmed cell death protein 5	14276.3	6	Cancer	
Q07812	BAX_HUMAN Apoptosis regulator BAX	21170.8	1	Cancer	
Q13153	PAK1_HUMAN Serine/threonine-protein kinase PAK 1	60608.8	4	Cancer	
O43464	*HTRA2_HUMAN Serine protease HTRA2, mitochondrial precursor*	48811	5		
O75340	*PDCD6_HUMAN Programmed cell death protein 6*	21854.8	103		
O95831	*AIFM1_HUMAN Apoptosis-inducing factor 1, mitochondrial precursor*	66859	57		
P55957	*BID_HUMAN BH3-interacting domain death agonist*	21981.1	7		
Q13158	*FADD_HUMAN Protein FADD*	23264.9	10		
Q8IX12	*CCAR1_HUMAN Cell division cycle and apoptosis regulator protein 1*	132738.6	72		
Q8N8D1	*PDCD7_HUMAN Programmed cell death protein 7*	54666.3	4		
Q96IZ0	*PAWR_HUMAN PRKC apoptosis WT1 regulator protein*	36545.5	5		
Q9BTC0	*DIDO1_HUMAN Death-inducer obliterator 1*	243720.8	76		
Q9BZZ5	*API5_HUMAN Apoptosis inhibitor 5*	57525.2	75		
Q9NR28	*DBLOH_HUMAN Diablo homolog, mitochondrial precursor*	27113.7	8		
Q9NYF8	*BCLF1_HUMAN Bcl-2-associated transcription factor 1*	106058.7	80		
Q9ULZ3	*ASC_HUMAN Apoptosis-associated speck-like protein containing a CARD*	21613.3	43		
**DNA Repair**					
O00255	MEN1_HUMAN Menin	67951	4	Cancer	
P09874	PARP1_HUMAN Poly [ADP-ribose] polymerase 1	113012.4	342	Cancer	Breast cancer
P12004	PCNA_HUMAN Proliferating cell nuclear antigen	28750.3	17	Cancer	
P12956	KU70_HUMAN ATP-dependent DNA helicase 2 subunit 1	69799.2	398	Cancer	Breast cancer
P13010	KU86_HUMAN ATP-dependent DNA helicase 2 subunit 2	82652.4	367	Cancer	Breast cancer
P16455	MGMT_HUMAN Methylated-DNA--protein-cysteine methyltransferase	21632.2	22	Cancer	Breast cancer
P18074	ERCC2_HUMAN TFIIH basal transcription factor complex helicase subunit	86854.3	3	Cancer	Breast cancer
P18887	XRCC1_HUMAN DNA-repair protein XRCC1	69483.1	15	Cancer	Breast cancer
P20585	MSH3_HUMAN DNA mismatch repair protein Msh3	127376.1	5	Cancer	
P27695	APEX1_HUMAN DNA-(apurinic or apyrimidinic site) lyase	35532.2	78	Cancer	Breast cancer
P29372	3MG_HUMAN DNA-3-methyladenine glycosylase	32842.8	36	Cancer	
P35244	RFA3_HUMAN Replication protein A 14 kDa subunit	13559.9	29	Cancer	
P43246	MSH2_HUMAN DNA mismatch repair protein Msh2	104676.8	21	Cancer	
P46063	RECQ1_HUMAN ATP-dependent DNA helicase Q1	73409.1	10	Cancer	
P49916	DNL3_HUMAN DNA ligase 3	102625.5	4	Cancer	Breast cancer
P52701	MSH6_HUMAN DNA mismatch repair protein MSH6	152688.4	37	Cancer	
P54727	RD23B_HUMAN UV excision repair protein RAD23 homolog B	43144.6	78	Cancer	Breast cancer
P78527	PRKDC_HUMAN DNA-dependent protein kinase catalytic subunit	468786.9	889	Cancer	Breast cancer
Q01831	XPC_HUMAN DNA-repair protein complementing XP-C cells	105915.3	14	Cancer	Breast cancer
Q12888	TP53B_HUMAN Tumor suppressor p53-binding protein 1	213440.8	7	Cancer	Breast cancer
Q92466	DDB2_HUMAN DNA damage-binding protein 2	47833.4	5	Cancer	
Q92878	RAD50_HUMAN DNA repair protein RAD50	153795.8	11	Cancer	Breast cancer
Q9UBB5	MBD2_HUMAN Methyl-CpG-binding domain protein 2	43228	7	Cancer	Breast cancer
Q9UHN1	DPOG2_HUMAN DNA polymerase subunit gamma-2, mitochondrial precursor	54876.3	7	Cancer	
P39748	*FEN1_HUMAN Flap endonuclease 1*	42566.1	114		
**Angiogenesis**					
P13489	RINI_HUMAN Ribonuclease inhibitor	49941.2	17	Cancer	
P40763	STAT3_HUMAN Signal transducer and activator of transcription 3	88011.4	13	Cancer	
Q16539	*MK14_HUMAN Mitogen-activated protein kinase 14*	41267.1	2		
Q14119	*VEZF1_HUMAN Vascular endothelial zinc finger 1*	56326.5	9		
Q13685	*AAMP_HUMAN Angio-associated migratory cell protein*	46721.5	1		
Q9UQB8	*BAIP2_HUMAN Brain-specific angiogenesis inhibitor 1-associated protein*	60829.7	5		
Q92793	*CBP_HUMAN CREB-binding protein*	265180.1	2		
**Migration/invasion/adhesion/metastasis**					
P07858	CATB_HUMAN Cathepsin B precursor	37796.8	6	Cancer	
P15941	MUC1_HUMAN Mucin-1 precursor	121999.6	4	Cancer	
P17931	LEG3_HUMAN Galectin-3	26172.1	77	Cancer	
P35222	CTNB1_HUMAN Catenin beta-1	85442.3	1	Cancer	
P62328	TYB4_HUMAN Thymosin beta-4	5049.5	15	Cancer	
Q06124	PTN11_HUMAN Tyrosine-protein phosphatase non-receptor type 11	68393.4	2	Cancer	
Q08380	LG3BP_HUMAN Galectin-3-binding protein precursor	65289.4	2	Cancer	
Q13153	PAK1_HUMAN Serine/threonine-protein kinase PAK 1	60608.8	4	Cancer	
Q13330	MTA1_HUMAN Metastasis-associated protein MTA1	80737.4	13	Cancer	Breast cancer
O60716	*CTND1_HUMAN Catenin delta-1*	108103.3	37		
P07339	*CATD_HUMAN Cathepsin D precursor*	44523.7	105		
P07355	*ANXA2_HUMAN Annexin A2*	38579.8	627		
P09493	*TPM1_HUMAN Tropomyosin alpha-1 chain*	32688.7	91		
P12814	*ACTN1_HUMAN Alpha-actinin-1*	102992.7	441		
P23528	*COF1_HUMAN Cofilin-1*	18490.7	508		
P35221	*CTNA1_HUMAN Catenin alpha-1*	100008.6	100		
P35580	*MYH10_HUMAN Myosin-10*	228796.8	136		
P60953	*CDC42_HUMAN Cell division control protein 42 homolog precursor*	21296.9	3		
P61586	*RHOA_HUMAN Transforming protein RhoA precursor*	21754.1	12		
P62258	*1433E_HUMAN 14-3-3 protein epsilon*	29155.4	136		
P63000	*RAC1_HUMAN Ras-related C3 botulinum toxin substrate 1 precursor*	21436.3	9		
Q07157	*ZO1_HUMAN Tight junction protein ZO-1*	195338.8	32		
Q13418	*ILK_HUMAN Integrin-linked protein kinase*	51386	43		
Q9UDY2	*ZO2_HUMAN Tight junction protein ZO-2*	133890.2	5		
Q13685	*AAMP_HUMAN Angio-associated migratory cell protein*	46721.5	1		
P08134	*RHOC_HUMAN Rho-related GTP-binding protein RhoC precursor*	21992.3	4		
**Differentiation**					
P15531	NDKA_HUMAN Nucleoside diphosphate kinase A	17137.7	154	Cancer	Breast cancer
P33076	C2TA_HUMAN MHC class II transactivator	123379.3	2	Cancer	
O60869	*EDF1_HUMAN Endothelial differentiation-related factor 1*	16358.9	9		
O95758	*ROD1_HUMAN Regulator of differentiation 1*	56466.5	15		
P09382	*LEG1_HUMAN Galectin-1*	14706.2	7		
P17931	LEG3_HUMAN Galectin-3	26172.1	77	Cancer	
P23771	*GATA3_HUMAN Trans-acting T-cell-specific transcription factor GATA3*	47885.3	3		
P35221	*CTNA1_HUMAN Catenin alpha-1*	100008.6	100		
P37231	PPARG_HUMAN Peroxisome proliferator-activated receptor gamma	57583.2	5	Cancer	Breast cancer
P40763	STAT3_HUMAN Signal transducer and activator of transcription 3	88011.4	13	Cancer	
Q8TB36	*GDAP1_HUMAN Ganglioside-induced differentiation-associated protein 1*	41225.8	2		
**Signaling**					
O00170	AIP_HUMAN AH receptor-interacting protein	37640.2	18	Cancer	Breast cancer
O14908	GIPC1_HUMAN PDZ domain-containing protein GIPC1	36026.7	34	Cancer	Breast cancer
P01112	RASH_HUMAN GTPase HRas precursor	21284.6	6	Cancer	Breast cancer
P02786	TFR1_HUMAN Transferrin receptor protein 1	84818	19	Cancer	Breast cancer
P08107	HSP71_HUMAN Heat shock 70 kDa protein 1	70009.2	560	Cancer	
P11142	HSP7C_HUMAN Heat shock cognate 71 kDa protein	70854.4	895	Cancer	
P15941	MUC1_HUMAN Mucin-1 precursor	121999.6	4	Cancer	
P16615	AT2A2_HUMAN Sarcoplasmic/endoplasmic reticulum calcium ATPase 2	114682.7	8	Cancer	
P29353	SHC1_HUMAN SHC-transforming protein 1	62812.6	8	Cancer	Breast cancer
P33076	C2TA_HUMAN MHC class II transactivator	123379.3	2	Cancer	
P34931	HS71L_HUMAN Heat shock 70 kDa protein 1 L	70331.5	482	Cancer	
P35222	CTNB1_HUMAN Catenin beta-1	85442.3	1	Cancer	
P37231	PPARG_HUMAN Peroxisome proliferator-activated receptor gamma	57583.2	5	Cancer	Breast cancer
P40763	STAT3_HUMAN Signal transducer and activator of transcription 3	88011.4	13	Cancer	
P78527	PRKDC_HUMAN DNA-dependent protein kinase catalytic subunit	468786.9	889	Cancer	Breast cancer
Q04206	TF65_HUMAN Transcription factor p65	60181.6	2	Cancer	
Q06124	PTN11_HUMAN Tyrosine-protein phosphatase non-receptor type 11	68393.4	2	Cancer	
Q07812	BAX_HUMAN Apoptosis regulator BAX	21170.8	1	Cancer	
Q13085	COA1_HUMAN Acetyl-CoA carboxylase 1	265382.7	1	Cancer	Breast cancer
Q13153	PAK1_HUMAN Serine/threonine-protein kinase PAK 1	60608.8	4	Cancer	
Q14653	IRF3_HUMAN Interferon regulatory factor 3	47189.7	2	Cancer	
Q15796	SMAD2_HUMAN Mothers against decapentaplegic homolog 2	52272.8	1	Cancer	
Q5JWF2	GNAS1_HUMAN Guanine nucleotide-binding protein G(s) subunit alpha isoforms XLas	110955.6	67	Cancer	Breast cancer
Q7LG56	RIR2B_HUMAN Ribonucleoside-diphosphate reductase subunit M2 B	40710.5	5	Cancer	
Q8WUF5	IASPP_HUMAN RelA-associated inhibitor	89036	15	Cancer	
Q92466	DDB2_HUMAN DNA damage-binding protein 2	47833.4	5	Cancer	
Q96RG5	Q96RG5_HUMAN Insulin receptor substrate 2 insertion mutant	137347.9	1	Cancer	Breast cancer
P31947	*1433S_HUMAN 14-3-3 protein sigma*	27756.7	67		
P62993	*GRB2_HUMAN Growth factor receptor-bound protein 2*	25190.4	32		
P46108	*CRK_HUMAN Proto-oncogene C-crk*	33810	18		
Q16539	*MK14_HUMAN Mitogen-activated protein kinase 14*	41267.1	2		
P42224	*STAT1_HUMAN Signal transducer and activator of transcription 1-alpha/be*	87279.6	62		
Q13547	*HDAC1_HUMAN Histone deacetylase 1*	55067.8	120		
Q92769	*HDAC2_HUMAN Histone deacetylase 2*	55328.8	31		
P56545	*CTBP2_HUMAN C-terminal-binding protein 2*	48914.2	26		
Q13363	*CTBP1_HUMAN C-terminal-binding protein 1*	47505.6	8		
Q92793	*CBP_HUMAN CREB-binding protein*	265180.1	2		
Q13616	*CUL1_HUMAN Cullin-1*	89622	12		
P62136	*PP1A_HUMAN Serine/threonine-protein phosphatase PP1-alpha catalytic subunit*	37487.8	179		
P62140	*PP1B_HUMAN Serine/threonine-protein phosphatase PP1-beta catalytic subunit*	37162.6	9		
P06493	*CDC2_HUMAN Cell division control protein 2 homolog*	34073.9	20		
P61586	*RHOA_HUMAN Transforming protein RhoA precursor*	21754.1	12		
P08134	*RHOC_HUMAN Rho-related GTP-binding protein RhoC precursor*	21992.3	4		
**Oxidative processes/redox**					
P00441	SODC_HUMAN Superoxide dismutase [Cu-Zn]	15925.9	35	Cancer	Breast cancer
P03891	NU2M_HUMAN NADH-ubiquinone oxidoreductase chain 2	38934.8	4	Cancer	Breast cancer
P04040	CATA_HUMAN Catalase	59718.9	3	Cancer	Breast cancer
P04179	SODM_HUMAN Superoxide dismutase [Mn], mitochondrial precursor	24706.6	1	Cancer	Breast cancer
P10599	THIO_HUMAN Thioredoxin	11729.7	28	Cancer	Breast cancer
P15559	NQO1_HUMAN NAD(P)H dehydrogenase [quinone] 1	30848	40	Cancer	Breast cancer
P16435	NCPR_HUMAN NADPH--cytochrome P450 reductase	76641.4	22	Cancer	Breast cancer
P21912	DHSB_HUMAN Succinate dehydrogenase [ubiquinone] iron-sulfur subunit	31608.9	5	Cancer	
P47985	UCRI_HUMAN Cytochrome b-c1 complex subunit Rieske, mitochondrial precursor	29649.4	2	Cancer	Breast cancer
Q99757	THIOM_HUMAN Thioredoxin, mitochondrial precursor	18371.6	1	Cancer	Breast cancer
Q07973	CP24A_HUMAN Cytochrome P450 24A1, mitochondrial precursor	58837.6	15	Cancer	Breast cancer
P30041	*PRDX6_HUMAN Peroxiredoxin-6*	25019.2	150		
P30044	*PRDX5_HUMAN Peroxiredoxin-5, mitochondrial precursor*	22012.5	110		
P30048	*PRDX3_HUMAN Thioredoxin-dependent peroxide reductase, mitochondrial precursor*	27675.2	48		
P32119	*PRDX2_HUMAN Peroxiredoxin-2*	21878.2	102		
Q06830	*PRDX1_HUMAN Peroxiredoxin-1*	22096.3	191		
**Various metabolic functions and DNA/RNA processing**					
O43708	MAAI_HUMAN Maleylacetoacetate isomerase	24166.7	4	Cancer	Breast cancer
P00390	GSHR_HUMAN Glutathione reductase, mitochondrial precursor	56221	1	Cancer	Breast cancer
P00492	HPRT_HUMAN Hypoxanthine-guanine phosphoribosyltransferase	24563.6	32	Cancer	
P00558	PGK1_HUMAN Phosphoglycerate kinase 1	44586.2	466	Cancer	
P07099	HYEP_HUMAN Epoxide hydrolase 1	52915	9	Cancer	Breast cancer
P11172	PYR5_HUMAN Uridine 5'-monophosphate synthase	52188.7	11	Cancer	
P11586	C1TC_HUMAN C-1-tetrahydrofolate synthase, cytoplasmic	101495.6	149	Cancer	Breast cancer
P15531	NDKA_HUMAN Nucleoside diphosphate kinase A	17137.7	154	Cancer	Breast cancer
P21266	GSTM3_HUMAN Glutathione S-transferase Mu 3	26542.2	59	Cancer	Breast cancer
P21964	COMT_HUMAN Catechol O-methyltransferase	30017.6	35	Cancer	Breast cancer
P23921	RIR1_HUMAN Ribonucleoside-diphosphate reductase large subunit	90012.5	4	Cancer	Breast cancer
P30876	RPB2_HUMAN DNA-directed RNA polymerase II subunit RPB2	133810.7	2	Cancer	
P34896	GLYC_HUMAN Serine hydroxymethyltransferase, cytosolic	53049.1	23	Cancer	Breast cancer
P35520	CBS_HUMAN Cystathionine beta-synthase	60548.3	9	Cancer	Breast cancer
P61604	CH10_HUMAN 10 kDa heat shock protein, mitochondrial	10924.9	58	Cancer	
P78417	GSTO1_HUMAN Glutathione transferase omega-1	27548	16	Cancer	Breast cancer
Q07973	CP24A_HUMAN Cytochrome P450 24A1, mitochondrial precursor	58837.6	15	Cancer	Breast cancer
Q13085	COA1_HUMAN Acetyl-CoA carboxylase 1	265382.7	1	Cancer	Breast cancer
Q7LG56	RIR2B_HUMAN Ribonucleoside-diphosphate reductase subunit M2 B	40710.5	5	Cancer	
Q7Z5J4	RAI1_HUMAN Retinoic acid-induced protein 1	203223.9	11	Cancer	
Q92820	GGH_HUMAN Gamma-glutamyl hydrolase precursor	35941.2	17	Cancer	
Q9UBB5	MBD2_HUMAN Methyl-CpG-binding domain protein 2	43228	7	Cancer	Breast cancer
Q9UHN1	DPOG2_HUMAN DNA polymerase subunit gamma-2, mitochondrial precursor	54876.3	7	Cancer	
**Cytoskeleton organization**					
P07437	TBB5_HUMAN Tubulin beta chain	49639	230	Cancer	Breast cancer
P62328	TYB4_HUMAN Thymosin beta-4	5049.5	15	Cancer	
Q13509	TBB3_HUMAN Tubulin beta-3 chain	50400.3	127	Cancer	Breast cancer
Q14980	NUMA1_HUMAN Nuclear mitotic apparatus protein 1	238113.2	505	Cancer	Breast cancer
Q92747	ARC1A_HUMAN Actin-related protein 2/3 complex subunit 1A	41542.7	1	Cancer	
**Transport/trafficking**					
O14908	GIPC1_HUMAN PDZ domain-containing protein GIPC1	36026.7	34	Cancer	Breast cancer
O75915	PRAF3_HUMAN PRA1 family protein 3	21600.4	19	Cancer	
Q8N1B4	VPS52_HUMAN Vacuolar protein sorting-associated protein 52 homolog	82169.8	14	Cancer

### Cell cycle regulation, proliferation and checkpoint

Among the key cell cycle and proliferation regulators, the cell cycle and mitotic checkpoint proteins with essential roles in maintaining the integrity of the cell division process (PRKDC, TP53BP1, BUB3, RB1), the proliferation markers (PCNA, KI-67, 14-3-3 sigma, PHB), the cyclin dependent kinases CDK2 and CDK1 (CDC2), the alpha and beta catalytic subunits of the protein phosphatase type 1 PP1 (PP1A and PP1B), and a series of other proteins that control transcription regulation, chromatin maintenance, mitosis, signaling and proteasome degradation, were identified. The phosphorylation of the RB1 protein by cyclin D1-CDK4/6 complexes plays an important role in cell advancement through the cell cycle and the regulation of the R-point: the unphosphorylated form is present in G0, hypophosphorylation correlates to entry into G1, and hyperphosphorylation is concurrent with passing of the restriction point and completion of the cell cycle. Upon exit from mitosis, the phosphate groups are removed by the Ser/Thr-protein phosphatase PP1 proteins [6]. Along with the cyclin-CDK complexes, protein phosphatases play an important role in cell cycle control through their modulation of signal transduction pathways. Active CDK2 is essential after the R-point, in late G1 and S, one of its roles in S being the phosphorylation of pol-α:primase which promotes DNA synthesis in S. Active CDK1 is essential in the M-phase, and also for entry into the S-phase in the absence of CDK2 [18]. Along with RB1, the guardian of the R-point gate, BUB3 acts as an M-phase mitotic spindle assembly checkpoint protein and inhibitor of the anaphase promoting complex (APC) that tags cell cycle proteins with ubiquitin for proteasomal degradation by the 26S proteasome [15]. TP53BP, through its association with p53, plays a key role in DNA damage response and transcription regulation, and PRKDC, a Ser/Thr kinase, in association with XRCC5/6 is a first-line responder and sensor of DNA damage. In parallel with their essential function in DNA repair, these proteins have additional roles in cell cycle regulation [12–15].

Consistent with cancer cell propagation, a number of known proliferation markers were detectable, i.e., PCNA, antigen Ki-67, 14-3-3 sigma and prohibitin-PHB [15, 19]. PCNA is involved in the control of eukaryotic DNA replication, and displays high expression levels in proliferating cells. Ki-67 is a marker of proliferation, being detectable in all stages of the cell cycle, except G0. 14-3-3 sigma is an adaptor protein which is involved in multiple signaling pathways, having a role in inhibiting G2/M cell cycle progression under p53 regulation. It modulates the activity of signaling proteins by binding to phospho-Ser/Thr motifs, and was found to be down-regulated in cancer cells. Prohibitin, on the other hand, inhibits DNA synthesis, and is believed to be a negative regulator of cell proliferation. Other players of the DNA replication machinery such as the origin replication complex (ORC) subunits 3, 4 and 5, the mini-chromosome maintenance (Mcm) proteins 2, 4 and 7, the replication protein A3 (RPA3 of RFA3) and flap endonuclease 1 (FEN1), as well as key proteins of the proteasomal degradation pathway, were detected. Protein degradation by the 26S proteasome pathway is an essential cell cycle regulatory process as it acts as a one-way switch that guarantees correct cell cycle phase transitions [20]. CUL1, a member of the Skp/Cullin/F-box complex (SCF) that controls the levels of CDK inhibitors p21 and p27, was also identified in the data set. Altogether, the data suggest that key positive and negative regulators of the cell cycle machinery are identifiable by MS in G1-arrested cells, and that these regulators act in various stages of protein synthesis and degradation, or protein alteration by posttranslational modifications.

### Apoptosis

Apoptosis is a tightly regulated process of cell destruction which cancer cells evade in a variety of ways, and this resistance has been recognized as a hallmark of tumorigenesis [16, 21]. The extrinsic pathway involves the activation of transmembrane death receptors from the tumor necrosis factor (TNF) superfamily (e.g., FAS and TNF-α) by death signals/ligands from the cell surface. Ligand binding results in the recruitment of FAS-associated death domain protein FADD which associates with procaspase-8 causing its activation and entry into the execution phase. The intrinsic pathway is triggered by a variety of stimuli such as growth factor withdrawal, hypoxia and direct DNA damage, among other factors, and acts through the p53 stress sensor. Once phosphorylated by DNA checkpoint proteins (ATM and CHK2), MDM2 mediated ubiquitination and tagging for proteolysis is impeded, and p53 proceeds to the activation of pro-apoptotic BCL-2 and repression of anti-apoptotic BCL-2 family of proteins. Increased p53 levels also lead to the increase of reactive oxygen species (ROS) that cause mitochondrial damage and release of DIABLO, ARTS and HTRA2 that activate the mitochondrial caspase cascade. The most relevant apoptosis protein markers that were identified included BAX (BCL-2 associated X protein which accelerates programmed cell death by binding to- and antagonizing apoptosis repressor BCL-2), AIFM1 (apoptosis-inducing factor 1), PDCD5/6/7 (with roles in induction and/or acceleration of apoptosis), DIDO1 (a programmed cell death protein), CCAR1 (a cell cycle and apoptosis regulator), ASC (a caspase-mediated apoptotic factor), HTRA2 (an inhibitor of the activity of inhibitors of apoptosis proteins), BID (BH3-interacting domain death agonist, a pro-apoptotic protein from the BCL-2 family), PAWR (a down-regulator of anti-apoptotic BCL-2), PAK1 (with roles in protection against apoptosis), API5 (an apoptosis inhibitor), and members of the m-TOR signaling pathway proteins with roles in cell survival and evasion of apoptosis [15, 21]. Beyond apoptosis, the activities of these proteins have broad ramifications into a multitude of signaling pathways including MAPK, ErbB and p53. BAX and PAK1 represent not only the most interconnected pro- and anti-apoptotic markers, but also provide a link between the DNA damage repair, proliferation and signaling protein clusters (see Figure 4).

### DNA damage response

Cells have developed various DNA repair mechanisms to correct for genomic damage caused by replication errors, chemical or environmental factors. The DNA damage repair proteins that were identified in G1 represent a large cluster of interacting proteins in Figure 4, and match the entire range of DNA damage response (DDR) pathways including mismatch repair (MSH), base excision repair (XRCC), nucleotide excision repair (XPC, PNKP, ERCC2), single and double strand break repair (XRCC, TP53BP, RAD50, PARP, PCNA, APEX, LIG3, PRKDC), homologous recombination and non-homologous endjoining [22]. A manifold of connections among all DNA repair proteins highlights the complex set of mechanisms that were developed by cells to preserve the genome integrity. Proteins with multiple interactions and roles such as PARP1 and PCNA are at the center of the DNA damage response network, and, as noted earlier for the apoptotic proteins, provide the functional link between the DNA damage, proliferation and cell cycle signaling clusters. As PARP1 has been found to be involved in the initiation of ssDNA break repair, and to play a major role in tumor development when dsDNA break repair cannot proceed via BRCA1/BRCA2 mediated homologus recombination due to various BRCA1/BRCA2 deficiencies, a variety of PARP inhibitors are under development for treating not just cancer, but also stroke and cardiovascular diseases [15].

### Angiogenesis

Angiogenesis is the highly regulated process of new blood vessel formation for the purpose of nutrient and oxygen supply required for cell function and survival [16]. Angiogenesis is a hallmark of cancer, but can be tied to other processes in the cell such as inflammation and wound healing [23]. G1-proteins with roles in angiogenesis included the gene products of MAPK14 (mitogen activated protein kinase), AAMP (angio-associated migratory cell protein), VEZF1 (vascular endothelial zinc finger), RNH1 (RINI) (ribonuclease/angiogenin inhibitor), STAT3 and p300/CBP (CREB-binding protein) [15]. MAPK14 can be activated by proinflammatory cytokines and through its kinase activity has roles in cell cycle regulation. AAMP has additional roles in cell migration and VEZF1 in transcription regulation and cellular defense response. RNH1 is an angiogenin inhibitor, STAT3 an angiogenesis modulator, while the p300/CBP proteins act as transcriptional co-activators of the angiogenic factor VEGF (vascular endothelial growth factor) [15, 16, 23]. Both pro- and anti-angiogenic factors possess various additional biological functions. MAPK14 and STAT3 lie at the heart of multiple signaling pathways that mediate gene expression in response to various stimuli, and play central roles in many cellular processes that link angiogenesis to cell growth, proliferation and apoptosis.

### Cell adhesion, migration, tissue invasion

Cell adhesion and migration play important roles in the initiation of tumor invasion and metastasis [24, 25]. Several tight junction (TJ) proteins, among which ZO1 and ZO2, and numerous adherens junction (AJ) proteins, which included α- and δ-catenin, were identified. Tight junctions (TJ) regulate the passage of ions and solutes between cells, while adherens junctions (AJ) participate in the initiation and stabilization of cell-cell contacts. Much broader roles in signal transduction, gene expression, cell cycle modulation and cytoskeleton regulation have been, however, described for these proteins. Transmembrane E-cadherin proteins use their extracellular domain to interact with E-cadherins on adjacent cells, and their intracellular domain to interact with p120-catenin (δ-catenin/CTND), α-catenin (CTNA) and β-catenin (CTNB). The catenins provide a link to the actin cytoskeleton and signaling pathways. Reduced levels of CTNA have been implicated in epithelial cancers, including breast cancer, and have been associated with increased invasiveness. CTND plays a role in the regulation of cell motility by interaction with RHO GTPases, and it has been implicated in breast cancer progression. On the cell surface, MUC1 (mucin), a membrane-bound O-glycosylated protein, plays additional roles in cell adhesion (the α-subunit) and the modulation of intracellular signaling pathways (the β-subunit) that include ERK, SRC, NF-kappa-B, RAS/MAPK and p53. Changes in the glycosylation pattern or overexpression of this protein have been frequently associated with carcinomas [26].

Cell motility involves cytoskeleton reorganization to extend the cell into the intended direction, as well as sever and create cell adhesions at the trailing and leading edges, respectively [27]. RHO, RAC and CDC42 are well studied cell motility regulators that belong to the RHO subfamily of RAS superfamily of GTPases [6]. Overexpression of these proteins has been linked to progression and metastasis of breast cancer. In addition, CATD, an estrogen regulated aspartyl protease that cleaves substrates such as fibronectin and laminin, has been associated with cell invasion and tumor invasiveness in breast cancer. TYB4 (with roles in actin polymerization), PTN11 (a protein tyrosine phosphatase involved in signaling) and LEG3 (carbohydrate binding) play additional roles in cell adhesion, migration and proliferation, while the expression of the MTA1 protein was correlated with metastatic potential [28]. PAK1, a Ser/Thr p21-activating kinase, is part of a family of proteins that link RHO GTPases to cytoskeleton reorganization and nuclear signaling, serving as targets for the small GTP binding proteins CDC42 and RAC [15]. It is known to regulate cell motility and morphology.

### Differentiation

Cell differentiation, proliferation and cell cycle regulation are processes that work concurrently, but independently, sharing certain key players with individual regulatory function [29]. Differentiation is the process by which unspecialized cells reach a terminal, non-proliferative state by acquiring structural and functional characteristics to perform a specific function [6]. Blocking of differentiation plays an important role in cancer pathogenesis, as poor cell differentiation correlates to more aggressive tumor phenotypes, and viceversa. There is evidence suggesting that the mechanisms that prevent hyperphosphorylation of RB favor differentiation, while mechanisms that promote RB hyperphosphorylation favor a block in differentiation [6]. Complementing the cell cycle and cell proliferation regulators (RB, MAPK, RAS-related proteins and protein phosphatases), a series of proteins with various roles in differentiation have been identified: GATA3, PPARG, EDF1, NME1 or NDKA, LGALS1 or LEG1, LGALS3, CTNNA1, GDAP1 and ROD1 [15]. GATA3, a transcriptional activator involved in the differentiation of luminal epithelial cells such as MCF-7 and that has been suggested as a breast cancer predictor, displays an inverse correlation to metastasis capability and strong association with estrogen receptors, though it does not appear to be involved in the estradiol signaling pathway [30]. In addition, GATA3 represses adipocyte differentiation by suppressing the peroxisome proliferator-activated receptor γ (PPARG). On the other hand, EDF1 (endothelial differentiation-related factor), a transcriptional activator, stimulates PPARG activities. The NME1, LGALS, CTNNA1 (catenin, alpha1), GDAP1 and ROD1 proteins have roles in inducing or blocking cell differentiation. The overlapping functional relationships between differentiation, invasive properties, angiogenesis and proliferation are clearly observable in Figure 4, and point in the direction of the same signaling proteins, strengthening the relevance of this protein set to determining cell fate.

### Oxidative phosphorylation/stress//redox regulation

Oxidative phosphorylation is an energy-producing metabolic pathway composed of five mitochondrial membrane-bound multiprotein complexes (I-V) which use the energy generated by electron transfer to synthesize ATP [31, 32]. A number of proteins belonging to NADH dehydrogenase, NADH ubiquinone oxidoreductase and ATP synthase complexes, cytochrome b-c complexes/oxidases, peroxiredoxins and thioredoxins, were identified. Aberrancies in oxidative phosphorylation such as electron leakage leading to oxidative stress, and mutations in these complexes, have been reported in cancer. Reactive oxygen species (ROS) formed from leaked electrons play a role in DNA damage and apoptosis, inducing oxidative stress, which in turn can promote angiogenesis and metastasis. Peroxiredoxins and antioxidant enzymes, such as [Cu-Zn] superoxide dismutase (SODC or SOD1, and SODM or SOD2) and catalase (CATA) modulate the levels of ROS and are important players in the cellular detoxification processes. While overproduction of ROS induces cell death, moderate levels reportedly can confer resistance to apoptosis and promote cell proliferation. Over expression of SOD1 and CATA has been reported in breast tumors and other types of cancer. APEX1, with roles in cell detoxification and the redox regulation of transcriptional factors, also displays activities in the base excision repair of DNA lesions induced by oxidative and alkylating agents where it functions as an apurinic/apyrimidinic endodeoxyribonuclease [15]. While this group of proteins formed a rather independent cluster of interacting partners in Figure 4 (see cluster 3), the link to the signaling and DNA damage response clusters is clearly evidenced through the superoxide radical capturing SOD1 and SOD2 proteins.

### Signaling

The largest sub-set of cancer markers included 48 proteins with various roles in modulating a broad range of signal transduction events (Table 2). Numerous pathways that promote cell proliferation are activated by the binding of various ligands to cell surface receptor tyrosine kinases (RTKs). SH2-containing proteins such as GRB2, SHC, STAT3 which bind phosphorylated RTKs, were present in the cytoplasmic fractions. GRB2, SHC1 and CRK are known as adaptor proteins because of their specific role as intermediates in protein-protein interactions [6, 16]. When GRB2 binds a phosphorylated RTK directly or through SHC1, it can initiate a signaling cascade by successive binding of SOS, a guanine nucleotide exchange factor that activates the membrane bound RAS protein by replacing GDP with GTP. Out of four existing mammalian RAS proteins, H-RAS was identified in the cytoplasmic fractions. RAS has a vast list of effector proteins which can activate different signaling pathways, of which the major ones are RAL-GDS, RAC/RHO, RAF, PI3K and RAL-GEF. The RAF mitogenic pathway has been described as possibly the most relevant to cancer pathogenesis due to its capability to activate several growth-promoting genes, provide anchorage independence, repress contact inhibition, change cell shape and in general promote proliferation [6, 16]. Downstream proteins in this pathway include the extracellular regulated protein kinases (ERK) ERK1 and ERK2. Pertinent to the G1/S transition, activation of ERK1 and ERK2 is required up to late G1 for expression of cyclin D1 and successful S entry, though the activity of these proteins is not necessary after the R-point. Further implications of RAS activation involve sustained angiogenesis and evasion of apoptosis. Overall, the deregulation of the SOS-RAS-RAF-MAPK signaling cascade is heavily implicated in acquired growth factor autonomy. In the larger landscape, RTK initiated signaling integrates ErbB, Jak/STAT, integrin, insulin, cell cycle/DNA repair and apoptosis signaling, chemokine signaling integrates Jak/STAT, G-protein and Ca signaling pathways, while Ras, downstream in the cell, further modulates the outcome of TGF-β, Wnt and NF-kB signaling. The ultimate result is a complex orchestration of cross-talk and positive/negative feed-back loops that control cell cycle progression.

**Table 2:** **Signaling pathways and associated proteins**

Pathway	Representative proteins
**MAPK:** Proliferation, differentiation, migration	HRAS/RASH, HSP71, HSP7C, HS71L, NF-kappa-β or TF65/RELA, GNAS, PAK1, RAC1, SHC1, MAPK14, GRB2, CRK
**ErbB:** Proliferation, differentiation, survival, angiogenesis and adhesion/motility/migration/invasion	SHC1, PAK1, GRB2, CRK, HRAS
**Cell cycle:** Proliferation	SMAD2, SHC1, 1433s/SFNC/stratifin, HDAC1/2, CREBBP, CUL1, CDC2
**DNA damage repair:** Maintenance of genome integrity	PARP1, TP53BP, PCNA, PRKDC, DDB2, CREBBP (Table 1)
**Apoptosis:** Cell death	BCLF1, BAX, TF65, IASPP
**p53:** Cell cycle arrest, apoptosis, senescence, inhibition of angiogenesis/metastasis, inhibition of IGF-1/mTOR pathway	BAX, RIR2B, DDB2, CDC2, 1433s
**TGF-β:** proliferation, apoptosis, differentiation, migration	SMAD2, CREBBP, CUL1, RHOA
**NF-kappa-B:** Regulation of genes involved in immunity, inflammation, cell survival	NF-kappa-β/TF65/RELA
**Wnt:** Cell division, development, adhesion	CTNB1, SMAD2, CTBP2, CREBBP, CUL1, RAC1
**Jak/STAT:** Growth, proliferation, development, cell fate, immunity, cell cycle, apoptosis	STAT 3, PTN11, STAT1, GRB2, CREBBP
**Toll-like:** Innate immune responses to pathogenic bacteria	TLR2, IRF3, TF65, MAPK14, STAT1, RAC1
**Notch:** Proliferative signaling, angiogenesis	HDAC1, CTBP1/2, CREBBP
**VEGF:** Angiogenesis	MAPK14, SHC1, RAC1, HRAS
**Adhesion/integrin signaling:** Cell migration, tissue invasion	CTNB1, PAK1, GRB2, CRK, HRAS, RAC1, PP1A/B, CDC2, RHOA, ILK, MUC1, PTN11
**Ca signaling:** Proliferation, apoptosis, metabolism	AT2A2, GNAS1
**Insulin signaling:** Maintenance of energy metabolism/homeostasis	Q96RG5, TFR1, PTN11, COA1, SHC1, GRB2, CRK, PP1A/B
**Chemokine:** Immune response, cell growth, differentiation, survival, migration, apoptosis, regulation of cytoskeleton	STAT3, PAK1, TF65, SHC1, GRB2, CRK, STAT1, RHOA, RAC1, HRAS
**PPAR signaling:** Lipid metabolism, adipocyte differentiation	PPARG, ILK
**Fatty acid biosynthesis**	COA1

### Discussion

**Figure 5:**
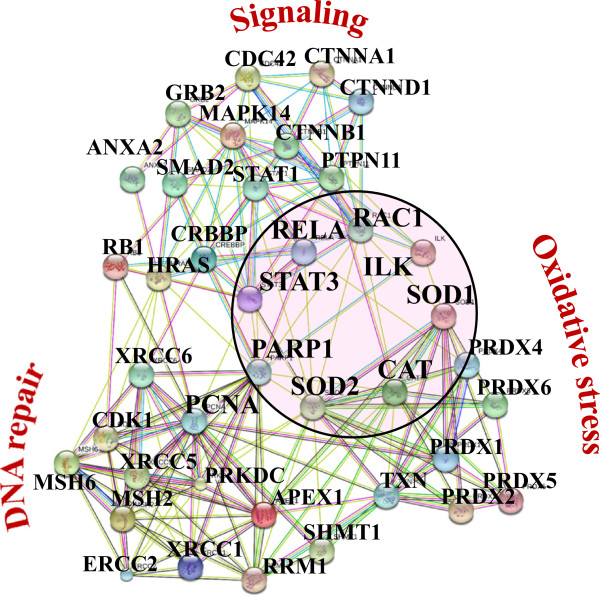
**STRING functional association network of central cancer markers that link the DNA repair, signaling and metabolic clusters.**

A first important finding of this study reveals that the majority of cancer marker proteins that were compiled in the DAVID disease database from rather random and unrelated studies on cancer, and that were identified in MCF-7 G1 cells, are not isolated players in the development of the disease, but part of multiple regulatory networks that integrate seamlessly with the hallmarks of cancer. A close examination of Figure 4 reveals that the proteins displaying the largest number of interactions (>10-15) also represent the functional links between the three major clusters: SOD1, SOD2 and CAT (from the oxidative stress cluster), PARP1, PCNA and APEX1 (from the DNA damage cluster) and STAT3, RAC1, RELA and ILK (from the signaling cluster). A STRING interaction diagram of this circle of proteins and of a few additional regulatory proteins with >5 interactors highlights in detail these central functional relationships (Figure 5). The outcome is conclusive: DNA damage co-exists with oxidative phosphorylation and stress, and, in response, multiple signaling pathways work in tandem to determine the fate of the cell. Cancer marker signaling proteins such as STAT3, RAC1, RELA and ILK are involved through their parent pathways essentially in all aspects of signal transduction (Table 2: Jak/STAT, NF-kappa-β, MAPK, Wnt, Toll-like, VEGF, chemokine, integrin), linking a vast array of extracellular stimuli to the intracellular signaling cascades that govern cell proliferation, repair, differentiation, immune response, invasion, metastasis or death. SOD2, SOD1 and CATA are key antioxidant defense enzymes that alleviate the toxic effects of hydrogen peroxide and superoxide anions/radicals produced as a result of various metabolic processes, while PARP1 and PCNA orchestrate DNA replication and damage repair functions to ensure healthy cell proliferation. Alterations in the activity of these genes and their protein products associate inherently, therefore, with the development of cancerous cell states. While members of all three major clusters were represented in both the nuclear and cytoplasmic cellular subfractions, the DNA damage repair components prevailed in the nuclear fraction, while the signaling and cellular detoxification components in the cytoplasm. Based on their functional roles, the nuclear markers and some of the signaling proteins are indicative of a network of drug targets, while the cytoplasmic proteins of a network or putative biomarkers, respectively. In recent years, the value of such a network-based set of markers has been recognized [33, 34]. New concepts such as network biomarkers and dynamical network biomarkers have gained popularity, primarily due to the promises brought to improving early diagnostics, sensitivity and specificity, and to behaving more robustly with smaller number of samples [33]. Moreover, the study of network models has suggested that therapies aimed at the inhibition of a number of drug targets, even if small and even if partial, can be much more effective than therapies aimed at the complete inhibition of a single target [34].

The second finding of this study reveals that a common thread of the identified cancer marker clusters is the presence of both agonist and antagonist members within the cluster. The results confirm that entire signaling pathways may have both proliferative and inhibitory outcomes. For example, the activation of STAT1 and STAT3 results in opposite effects on tumorigenesis. While STAT3 is considered an oncogene that promotes cell survival/proliferation, motility and immune tolerance, STAT1 is a tumor suppressor through its anti-proliferative, pro-apoptotic and angiogenesis-inhibitor activities [23]. The active TGF-β signaling pathway has, on the other hand, a role in growth inhibition. Upon activation of the pathway, SMAD2 and SMAD3 undergo phosphorylation in the cytosol followed by binding of either phosphorylated protein to SMAD4, nuclear translocation of the complex, and transcription factor activity. Relevant to cell cycle control, the CDK inhibitors p15 and p21 are the important targets of this pathway [6]. Repression of TGF-β signaling is therefore a way in which cancer cells can achieve insensitivity to antigrowth signals. For example, EVI1 and AML1/EVI1 inhibit the transcription factor activity of SMAD3 in the nucleus by direct interaction through a zinc-finger motif. Furthermore, CTBP (C-terminal binding protein), a transcriptional repressor necessary for the inhibition of SMAD3 by EVI1, recruits a histone deacetylase (HDAC) complex which aids in the repression of antigrowth signals [24]. The two CTBP vertebrate homologues (CTBP1 and CTBP2), as well as the histone deacetylases HDAC1 and HDAC2, were identified in the nuclear fractions, suggesting that important participants of acquisition of insensitivity to antigrowth signals are in place in MCF-7 cells. For a complex disease such as cancer, that hosts entirely deregulated but viable signaling pathways, the identification of a novel regulatory network such as suggested by Figures 4 and 5, of its components and of its dynamic behavior, is expected to have a major impact on clarifying the mechanistic details of disease progression.

The third finding of the study reveals that the identified signaling clusters control or modulate not one, but several cancer-related biological processes. Likewise, physiological responses within a cell are elicited not through one, but through multiple signaling pathways. The MAPK and Jak/STAT pathways have ramifications within, virtually, all biological processes that determine the fate of a cell. The delineation of a panel of proteins that modulate cell adhesion/motility/metastasis (CTNB1, MUC1, PAK1, RAC1, RHOA, PTN11, CRK, GRB2, PP1A/B), while simultaneously playing central roles in signaling pathways such as MAPK, ErbB, IGF and Jak/STAT (and, as a result, in all aspects of cell division, proliferation, apoptosis and differentiation, see Tables 1 and 2), is vital to providing insights into the mechanism used by cancer cells to invade adjacent tissues and into the correlation of these processes with events occurring within the cytoplasmic signal-transduction pathways. Simultaneously, the placement of both pro- and anti-apoptotic proteins within a broader panorama of cell cycle regulation, survival, proliferation and differentiation proteins is critical to exploring the impact of anti-apoptotic factor up-regulation or pro-apoptotic factor down-regulation in cancer. Such perspectives can reveal clues into whether cancer cells have (or not) the ability to activate self-destruction signaling mechanisms, and into the strategies evolved by cancer cells to evade apoptosis and respond to cellular signals that indicate a malfunction of the cell proliferation machinery or the presence of physiological stress. Future protein differential expression studies will be able to validate which networks prevail in the case of particular pathological cancer phenotypes.

## Conclusions

In this work, through proteomic profiling of the G1 stage of MCF-7 cells, and by making use of publically available information and bioinformatics tools, we uncovered a highly interconnected network of nuclear and cytoplasmic cancer markers with regulatory role in biological processes representative of all hallmarks of cancer. Protein interaction analysis and biological characterization of the data revealed that clusters pertaining to cell cycle regulation, signaling, DNA repair, differentiation, angiogenesis and apoptosis, were particularly well represented in the pool of identified proteins. The three major networks formed by these cancer markers, i.e., signaling, maintenance of genome integrity and oxidative stress, are indicative of the different mechanisms that cancer cells utilize to maintain viability in the absence of mitogenic stimulation and to possibly evade the R-restriction point and sustain an aberrant proliferative status. Due to their collective and intertwined roles on the proliferative behavior of cancer cells, the identified markers represent a panel of broad functional relevance to both biomarker and drug discovery research. The combined biomarker/drug-target potential is elevated by the fact that the panel emerged from a list of markers representative of a number of cancerous cell states, compiled rather randomly by on-line bioinformatics tools. The data suggest that proteins with redundant or multiple roles in the control of a cell’s fate represent the most informative cancer markers and the most valuable leads for the development of multiplexed biomarker assays and of anti-cancer drugs with increased therapeutic potential.

## Electronic supplementary material

Additional file 1: **Title of data: MCF-7 G1 cell cycle proteins and DAVID functional clustering.** Description of data: The table contains proteins identified in the MCF7 G1 stage of the cycle and their DAVID categorization. (XLSX 710 KB)

Additional file 2: **Title of data: List of cancer markers with associated biological and mass spectrometry information.** Description of data: The table contains proteins identified in the MCF7 G1 stage of the cycle that were associated with the presence of cancer, their GO categorization, disease associations, and Kegg pathways. (XLSX 2 MB)
